# Genotype-Specific Amino Acid Signatures in the Gn/Gc Glycoprotein of Severe Fever with Thrombocytopenia Syndrome Virus Associated with Neurological Symptoms

**DOI:** 10.3390/pathogens15090946

**Published:** 2026-09-06

**Authors:** Qiongbin Mao, Bohan Zhang, Haowen Yuan, Zhongru Zhao, Jingwan Han, Hanping Li, Yongjian Liu, Lei Jia, Xiaolin Wang, Hongling Wen, Lin Li

**Affiliations:** 1 Department of Health Inspection and Quarantine, School of Public Health, Cheeloo College of Medicine, Shandong University, Jinan 250012, China; maoqiongbin982001@163.com (Q.M.); yuanhaowen0218@163.com (H.Y.); zhaozhongru@foxmail.com (Z.Z.); 2 Shandong Provincial Key Laboratory for Prevention and Control of Emerging Infectious Diseases and Biosafety in Higher Education Institutions, Jinan 250012, China; 3 State Key Laboratory of Pathogen and Biosecurity, Academy of Military Medical Sciences, Beijing 100071, China; zbhforjob@163.com (B.Z.); hanjingwan@outlook.com (J.H.); hanpingline@163.com (H.L.); yongjian325@sina.com (Y.L.); 15001193408@163.com (L.J.); woodsxl@163.com (X.W.); 4 Shandong Provincial Key Laboratory of Intelligent Monitoring, Early Warning, Prevention and Control for Infectious Diseases, Jinan 250012, China; 5 Yellow River National Strategy Institute, Shandong University, Jinan 250012, China

**Keywords:** SFTSV, Gn/Gc glycoprotein, neurological symptoms, M segment-based genotyping, genotype-specific marker, viral genomic epidemiology

## Abstract

Severe fever with thrombocytopenia syndrome (SFTS) is a highly fatal tick-borne disease caused by SFTS virus (SFTSV). The Gn/Gc glycoprotein plays a critical role in viral entry, but whether its amino acid polymorphisms directly determine neurological symptoms remains unclear. We sequenced the M segment from 99 SFTS patients (31 with neurological symptoms, 68 without) in Shandong Province, China. After removing recombinant sequences, 91 non-recombinant sequences were analyzed and genotyped based on M segment phylogeny. Systematic signature screening, phylogenetic reconstruction, genotype-stratified analysis, public database validation, cross-genotype comparison, and selection pressure inference were applied to distinguish functional determinants from genotype-specific markers. M segment-based phylogeny classified the sequences into four genotypes (D, E, F, A), with D accounting for 79.3% of NS cases. Although 10 candidate sites initially distinguished NS from Non-NS, their associations disappeared upon genotype-stratified analysis; all were low-frequency variants (<15%) in public databases. Cross-genotype comparison revealed D-specific (“GERHVGIIFL”) and E/F-specific (“DGKQMRVTSF”) patterns, none of which overlapped with positively selected sites. These findings demonstrate that the apparent NS association reflected genotype confounding rather than functional determinants, identifying the 10 sites as potential genotype D-specific markers with high discriminatory potential, warranting further validation in independent datasets. This study provides a cautionary example for virus–phenotype association studies, emphasizing that genotype stratification is essential before interpreting amino acid differences as functional.

## 1. Introduction

Severe fever with thrombocytopenia syndrome (SFTS), a tick-borne disease with fatality rates between 5% and 30%, is caused by the SFTS virus (SFTSV) [1]. First identified in rural areas of Hubei and Henan provinces in China in 2009, SFTS has since become endemic across East Asia, with confirmed human cases reported in South Korea, Japan, Thailand, and Vietnam [2]. In 2024, the World Health Organization (WHO) listed SFTSV as a priority pathogen, reflecting its broad geographic distribution, increasing incidence, and the absence of approved vaccines or effective antiviral therapies [3].

SFTSV is a segmented negative-strand RNA virus belonging to the genus Bandavirus in the family Phenuiviridae [4]. Its genome consists of three segments—large (L), medium (M), and small (S)—encoding the RNA-dependent RNA polymerase (RdRp), the glycoprotein precursor (GP) that is post-translationally cleaved into Gn and Gc, the nucleoprotein (NP), and the non-structural protein (NSs) [5]. Among these, the Gn and Gc glycoproteins are the only envelope proteins on the viral surface. They form heterodimers that constitute the viral spike structures, mediating host cell receptor recognition, attachment, and membrane fusion, making them primary determinants of viral tissue tropism and pathogenicity, as well as the main targets of neutralizing antibodies [6]. Given their critical roles in viral entry and immune evasion, genetic variation in Gn/Gc may profoundly influence viral fitness and clinical outcomes.

Phylogenetically, SFTSV is classified into genotypes A–F, with genotype D predominating in Shandong Province and associated with higher case fatality rates and neurological symptoms, whereas genotypes E and F are more common in Japan, South Korea, and Zhejiang Province [7]. Genotype D has also been linked to elevated case fatality rates and unique linkage networks [8]. Recently, a deeply divergent novel genotype (provisionally designated genotype G) was identified in northern Hebei Province, dating its origin to the late 13th century—over 400 years before genotypes A–F emerged [9]. This discovery substantially expands the known genetic diversity of SFTSV and underscores the complexity of its evolutionary history in East Asia.

Clinical outcomes after SFTSV infection vary greatly, ranging from mild febrile illness to severe disease with hemorrhagic manifestations, encephalitis, and multiple organ dysfunction. Clinically, encephalitis occurs in approximately 19% of hospitalized patients and is associated with a 44.7% case fatality rate, and neurological symptoms are a strong independent predictor of disease progression (OR = 8.244), along with age, C-reactive protein, D-dimer, and viral load [10,11,12]. SFTSV isolation from cerebrospinal fluid confirms central nervous system invasion, but the molecular basis of this neurotropism remains unclear. In particular, whether specific Gn/Gc amino acid polymorphisms facilitate viral entry into neural tissues or modulate host inflammatory responses that lead to neurological impairment has not been systematically investigated.

Despite substantial progress in understanding SFTSV epidemiology and genetic diversity, the molecular basis of neurological symptom variation remains elusive. While genotype-associated differences in clinical outcomes have been observed, it remains unclear whether the Gn/Gc polymorphisms that distinguish these genotypes are direct functional drivers of neuropathogenesis or merely lineage-specific markers that correlate with neurological symptoms through genotype distribution bias. Resolving this ambiguity is critical for understanding SFTSV pathogenesis and for distinguishing functional determinants from genotype-associated background polymorphisms.

In this study, we sequenced the M segment of SFTSV from 99 patients with and without neurological symptoms in Shandong Province and performed systematic amino acid signature screening to identify residues distinguishing the two clinical phenotypes. Through phylogenetic reconstruction based on M segment sequences, genotype-stratified analysis, cross-genotype comparison, public database validation, and selection pressure inference, we systematically evaluated the candidate sites. The stark clinical contrast between genotype D and F strains in our patient series provided a unique opportunity to distinguish genuine functional determinants from genotype-specific markers and to establish a methodological framework for interpreting genotype-phenotype associations in viral genomic epidemiology.

## 2. Materials and Methods

### 2.1. Study Population and Sample Collection

This retrospective study enrolled 99 SFTS patients (31 NS, 68 Non-NS) diagnosed at Shandong Provincial Public Health Clinical Center between January 2020 and December 2025. The diagnosis of SFTS was confirmed by real-time RT-PCR detection of SFTSV RNA in serum, following the Chinese Ministry of Health criteria. Neurological symptoms (NS) were defined as acute onset with fever, headache, or vomiting accompanied by consciousness disturbance (lethargy, confusion, seizures, or coma) or meningeal signs. Patients with pre-existing neurological or psychiatric disorders were excluded to avoid confounding, based on documented medical histories recorded in the admission notes and previous medical records. Demographic and clinical data, including age, sex, onset-to-admission time, underlying diseases, and laboratory parameters on admission (white blood cell count, platelet count, aspartate aminotransferase, and alanine aminotransferase), were extracted from medical records.

### 2.2. RNA Extraction, Sequencing, and Recombination Analysis

Total RNA was extracted from 200 μL of serum using the High Pure Viral RNA Kit (Roche, Basel, Switzerland) according to the manufacturer’s instructions. Briefly, 200 μL of serum was mixed with 400 μL of binding buffer containing poly(A) carrier RNA and proteinase K, incubated at room temperature for 10 min, and then loaded onto the High Pure filter tube. After centrifugation and wash steps, RNA was eluted in 70 μL of elution buffer and either used immediately for cDNA synthesis or stored at −80 °C.

The full-length M segment was amplified by nested RT-PCR with four overlapping primer pairs (Table S1). First-round PCR (50 μL) contained 2× One Step Buffer, PrimeScript 1 Step Enzyme Mix, external primers (10 μmol/L each), and 5 μL RNA template, using PrimeScript One Step RT-PCR Kit Ver.2 (Takara, Shiga, Japan). Cycling conditions were 50 °C for 30 min (reverse transcription), 95 °C for 2 min, followed by 35 cycles of 95 °C for 30 s, 58 °C for 30 s, and 72 °C for 45 s, with a final extension at 72 °C for 5 min. The nested second-round PCR used DreamTaq Green PCR Master Mix (2×, Thermo Scientific, Waltham, MA, USA) with 5 μL of first-round product and internal primers (10 μmol/L each) in a 50 μL reaction, under the same thermal profile.

PCR products were purified using the Wizard SV Gel and PCR Clean-Up System (Promega, Madison, WI, USA) and quantified using a Qubit 4 fluorometer (Thermo Fisher, Waltham, MA, USA) with the dsDNA HS Assay Kit (Thermo Fisher, Waltham, MA, USA). Equimolar amounts of each purified fragment were pooled to generate a single mixed DNA sample. Using a Covaris instrument (Covaris, Woburn, MA, USA), the pooled amplicons (roughly 800–1000 bp) were sheared down to a uniform size range of 250–300 bp.

For library preparation, we followed the NEB Next Ultra DNA Library Prep Kit for Illumina (New England Biolabs, Ipswich, MA, USA) guidelines. Briefly, the fragmented DNA underwent end repair and A-tailing before ligation to complete Illumina adapters. The resulting ligation products were then PCR-amplified and cleaned up with AMPure XP beads (Beckman Coulter, Brea, CA, USA). Library quality and fragment size distribution were assessed using an Agilent 2100 Bioanalyzer (Agilent Technologies, Santa Clara, CA, USA), and libraries were quantified by real-time PCR. Sequencing was performed on the Illumina NovaSeq 6000 platform (Illumina, San Diego, CA, USA) with 150 bp paired-end reads (PE150). Cluster generation was performed on a cBot system using the Illumina PE Cluster Kit (Illumina, San Diego, CA, USA), and sequencing by synthesis (SBS) was conducted according to the manufacturer’s protocols. Low-titer samples were sequenced on the same platform to ensure adequate coverage.

We filtered the raw reads to remove those carrying adapters, containing more than 10% ambiguous nucleotides, or having over 50% of bases with a Phred score below 5. High-quality reads were assembled with CLC Genomics Workbench (v25) and aligned using MAFFT (v7.487) with the L-INS-i strategy [13]. Reference sequences for genotypes A–G were downloaded from GenBank. Recombination analysis was performed using RDP5(v5.84) [14] with seven detection algorithms (RDP, GENECONV, BootScan, MaxChi, Chimaera, SiScan, and 3Seq), a sliding window of 200 bp, a step size of 20 bp, and Bonferroni-corrected *p* < 0.05. Recombination events supported by at least four algorithms were retained; SimPlot was used to validate breakpoints. Recombinant sequences were excluded from downstream analyses.

### 2.3. Phylogenetic Analysis

Phylogenetic reconstruction was performed using the maximum likelihood method in IQ-TREE (version 3.0.1) [15], where ModelFinder selected the optimal nucleotide substitution model by evaluating the Bayesian Information Criterion (BIC). Branch support was assessed via ultrafast bootstrap with 1000 replicates. Reference sequences encompassing genotypes A–G were included, with OP313000.1 representing genotype G and three sequences (PV446566.1, PV446563.1, PV446560.1) designated as outgroups. Bootstrap values ≥ 70% were considered robust. Trees were visualized and annotated using iTOL (version 6) [16].

### 2.4. Signature Site Screening

Amino acid positions were numbered from the initial methionine (+1) using HB29 as reference. Signature pattern analysis was performed using an entropy-based algorithm that ranks positions by discriminatory power without a fixed frequency threshold [17], identifying a collective signature pattern across multiple positions rather than relying on individual site discrimination. For candidate prioritization, VESPA-identified sites were further filtered using our predefined criteria: (i) characteristic amino acid frequency ≥ 50% in the NS group and ≥50% alternative in the Non-NS group, with Fisher‘s exact *p* < 0.05; or (ii) high discriminatory weight in the VESPA output. Sites meeting either criterion were retained for validation.

### 2.5. Genotype-Stratified Analysis

To exclude genotype confounding and assess whether the candidate sites were genuinely associated with neurological symptoms or merely reflected genotype background, we restricted the analysis to genotype D sequences (NS: 23, Non-NS: 28). For each of the 10 candidate sites, the frequency of the NS characteristic amino acid was recalculated within genotype D, and Fisher’s exact test was used to compare frequencies between the NS and Non-NS groups. A *p*-value < 0.05 was considered statistically significant. A genome-wide scan across all 1073 amino acid positions was also performed within genotype D using the same signature pattern analysis criteria as described in Section 2.4, retaining sites with Fisher’s exact *p* < 0.05, to identify any potential intra-genotype associations with neurological symptoms.

### 2.6. Public Database Validation

The natural frequency of the candidate amino acid residues in the global SFTSV population was assessed by retrieving glycoprotein precursor (GP) sequences from the NCBI GenPept database using the taxonomy IDs for Severe fever with thrombocytopenia syndrome virus (Taxonomy ID: 1003835) and Bandavirus dabieense (Taxonomy ID: 2748958). Sequences were filtered to retain only full-length GP proteins with a length of 1000–1100 amino acids. After deduplication, 2219 unique sequences were retained for further analysis. For each candidate site, the frequency of the NS-characteristic amino acid was calculated across the dataset, and residues with a frequency below 15% were defined as low-frequency variants.

### 2.7. Cross-Genotype Comparison

The distributions of NS characteristic amino acids were compared across genotypes D, E, and F to determine whether the 10 candidate sites were genotype-specific markers rather than symptom-associated sites. The characteristic amino acid pattern of each genotype was determined by deriving the consensus sequence at the 10 candidate positions using the most frequent residue at each position within each genotype group. Sensitivity and specificity of the 10-site combination as a genotype D-specific marker were calculated against a curated reference sequence set. Sensitivity was calculated as TP/(TP + FN), and specificity as TN/(TN + FP), where TP, FN, and TN were defined based on exact matching of the genotype D-specific consensus pattern. All calculations used reference sequences downloaded from GenBank.

We next examined the internal linkage among these 10 sites within genotype D. Co-occurrence network analysis was performed using Fisher’s exact test on the 51 genotype D sequences. For each pair of the 10 VESPA-identified sites, we tested whether the genotype D-characteristic amino acids co-occurred. All 45 pairs were significant (*p* < 0.05; ranging from 1.67 × 10^−26^ to 7.80 × 10^−11^), resulting in a fully connected network with a density of 1.000. No multiple testing correction was applied for this exploratory co-occurrence network analysis, as it was designed to describe the linkage structure within genotype D rather than for confirmatory hypothesis testing.

### 2.8. Selection Pressure Inference

Genome-wide selection pressure analyses were performed independently for the NS (*n* = 29) and Non-NS (*n* = 62) groups using four complementary algorithms via HyPhy on the Datamonkey server (https://www.datamonkey.org/) [18,19] to assess whether the candidate sites were subject to adaptive evolution. Global selection pressure was estimated using FUBAR (Fast Unconstrained Bayesian AppRoximation), with sites having a posterior probability > 0.9 considered positively selected. The global dN/dS (ω) values for each group were extracted from the FUBAR output. Site-specific positive selection was inferred with MEME (Mixed Effects Model of Evolution), a method that scans for episodic selection along distinct phylogenetic lineages. The significance cut-off was set at *p* < 0.1. Cross-validation was performed using SLAC (Single-Likelihood Ancestor Counting) and FEL (Fixed Effects Likelihood) with the same significance threshold. Sites identified by at least two methods were classified as robust positively selected sites [18,19].

### 2.9. Statistical Analysis

Statistical analyses were performed using R (version 4.3). Continuous variables were compared with Wilcoxon rank-sum tests, and categorical variables with Fisher’s exact tests. Univariate logistic regression was initially used to screen for predictors of neurological symptoms, but the exclusive presence of genotype F in the Non-NS group caused complete separation, making multivariable odds ratio (OR) estimates unreliable. Accordingly, genotype–phenotype associations were assessed using Fisher’s exact tests. As a sensitivity analysis for complete separation, we also performed Firth’s penalized logistic regression using the logistf package in R. A two-sided α = 0.05 was used for all tests. During manuscript preparation, the authors used DeepSeek solely for language polishing and grammar improvement, and take full responsibility for the final content.

## 3. Results

### 3.1. Clinical Characteristics and Phylogenetic Analysis

**Figure 1:**
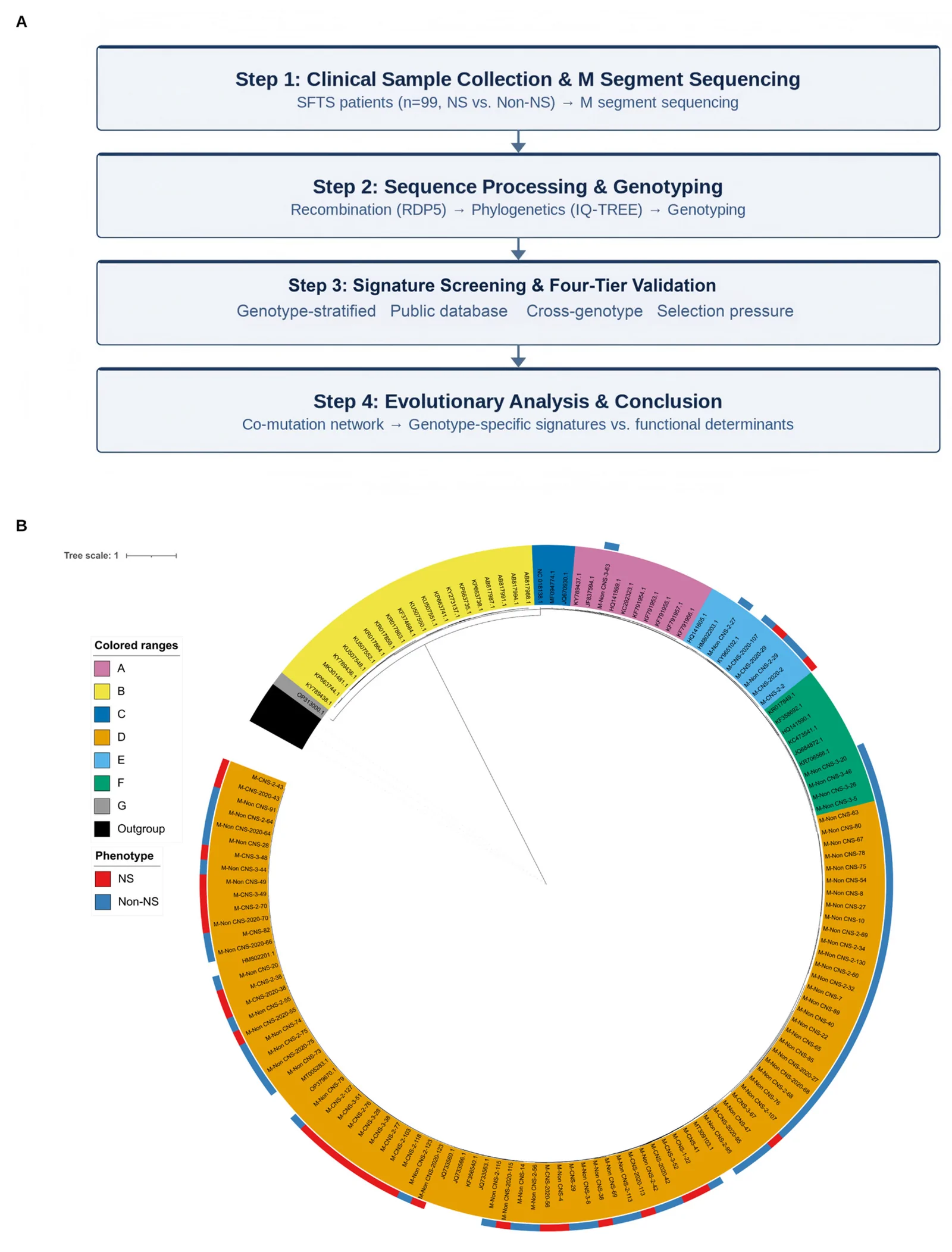
Study framework and phylogenetic background. (**A**) Schematic overview of the study workflow. M segment sequencing was performed on 99 SFTS patients, followed by recombination analysis, phylogenetic reconstruction, genotyping (based on M segment sequences), signature screening, and sequential validation steps (genotype-stratified analysis → public database validation → cross-genotype comparison → selection pressure inference). (**B**) Maximum likelihood phylogenetic tree of 91 non-recombinant M segment sequences with 53 reference sequences (genotypes A–G). NS and Non-NS sequences are indicated by colored rings on the outer track (red: NS; blue: Non-NS). Sequences from both groups are scattered among different genotypes, with no lineage specifically associated with symptoms. Nodes with bootstrap support greater than 75% are marked. The scale bar represents 0.01 nucleotide substitutions per site.

A total of 99 M-segment sequences were obtained. RDP5 identified eight recombinant sequences (8.1%), with inter-genotype recombination accounting for 87.5% (7/8). Genotype D participated in six recombination events, and recombination breakpoints clustered at the 5’ end (55–815 bp, corresponding to Gn) and the 3′ end (2209–3237 bp, corresponding to Gc) (Figure S1). Comparison of recombinant versus non-recombinant sequences showed comparable clinical and genotypic profiles (Table S2), confirming that exclusion of recombinant sequences did not systematically bias subsequent analyses. Parental genotype analysis revealed that genotype D was involved in seven of the eight recombinant sequences (87.5%), genotype F in five (62.5%), and genotype E in three (37.5%). Following recombination, the eight sequences were assigned to final genotypes as: five (62.5%) genotype D, two (25.0%) genotype F, and one (12.5%) genotype E. Among these, one presented with neurological symptoms (12.5%). After excluding recombinant sequences, 91 non-recombinant sequences (29 NS, 62 Non-NS) were retained for downstream analyses as outlined in the study workflow (Figure 1A).

As shown in Table 1, NS patients were significantly older than Non-NS patients (median 70 vs. 67 years, *p* = 0.042), consistent with previous reports identifying advanced age as a risk factor for SFTS-associated encephalitis. No significant differences were observed between the two groups in sex distribution, time from symptom onset to admission, underlying diseases, or laboratory parameters on admission (white blood cell count, platelet count, aspartate aminotransferase, and alanine aminotransferase; all *p* > 0.05), indicating that the two groups were well balanced for these baseline clinical characteristics. To further assess whether the association between genotype D and neurological symptoms was independent of host demographic factors, we performed multivariable logistic regression adjusting for age and sex. After adjustment, age showed a marginal association with neurological symptoms (OR = 1.06 per year, 95% CI: 0.99–1.15, *p* = 0.067), while sex was not significantly associated (OR = 1.42, 95% CI: 0.48–4.39, *p* = 0.531). Within genotype D, the median age of NS patients (71.5 years, IQR: 67–75.8) was not significantly different from that of Non-NS patients (69 years, IQR: 66–73; *p* = 0.230, Wilcoxon rank-sum test), suggesting that the overall age difference between the two groups in the full cohort was likely attributable to genotype distribution rather than representing an independent confounding factor.

**Figure 2:**
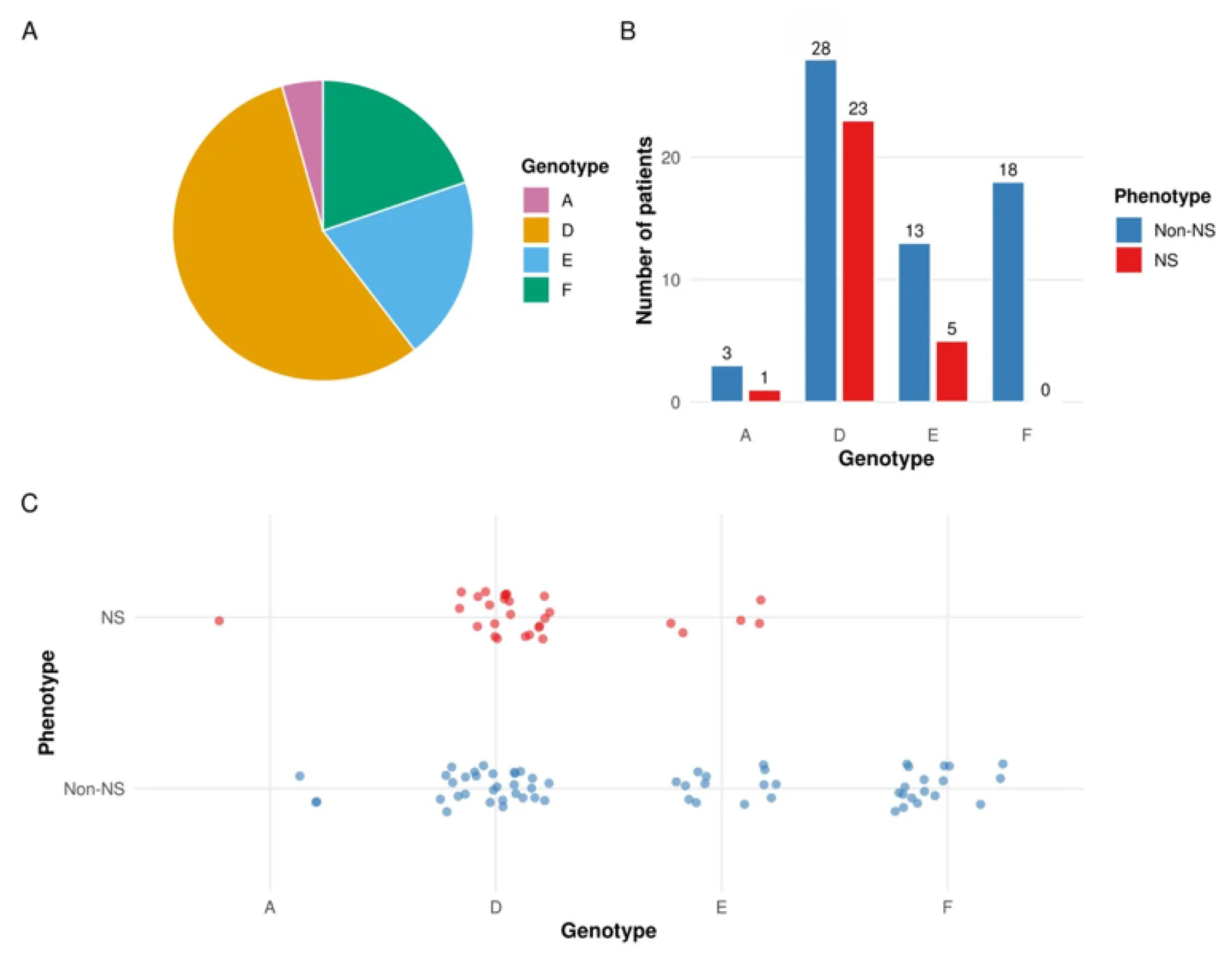
Genotype distribution and association with neurological symptoms. (**A**) Pie chart showing M segment-based genotype distribution of 91 non-recombinant SFTSV sequences. Genotype D (56.0%) was predominant, followed by genotypes E (19.8%) and F (19.8%). (**B**) Bar chart showing the proportion of neurological symptom cases within each genotype. Among genotype D strains, 45.1% (23/51) were from the NS group, whereas none of the genotype F strains (0/18) were from the NS group (*p* = 0.0003, Fisher’s exact test). (**C**) Dot plot showing the distribution of neurological symptom cases across genotypes. Each dot represents an individual patient; red indicates NS and blue indicates Non-NS.

We next performed phylogenetic analysis to determine the genotype distribution of the infecting SFTSV. As shown in Figure 1B, the 91 non-recombinant sequences belonging to NS and Non-NS groups intermingled within major genotype branches, with no neurological symptom-specific lineage observed. To characterize the genotype distribution, we classified the 91 non-recombinant sequences into four M segment genotypes: D (51, 56.0%), E (18, 19.8%), F (18, 19.8%), and A (4, 4.4%) (Figure 2A). Notably, all genotype F strains were exclusively identified in the Non-NS group (18/18), showing complete separation from the NS group (*p* = 0.0003). This indicates a statistical association with the absence of neurological symptoms, but the effect size cannot be reliably estimated due to complete separation. In contrast, genotype D accounted for 79.3% (23/29) of NS cases in the non-recombinant dataset. The incidence of neurological symptoms was 45.1% (23/51) for genotype D compared with 0% (0/18) for genotype F (Figure 2B,C). As a sensitivity analysis to address the complete separation issue, Firth’s penalized logistic regression further supported this association (Firth OR = 0.086, 95% CI: 0.009–0.383, *p* = 0.0005). This stark clinical contrast between genotype D and F strains provided the initial rationale for investigating whether specific Gn/Gc amino acid polymorphisms could explain the differential neurological risk.

### 3.2. Identification of Candidate Amino Acid Signatures

**Figure 3:**
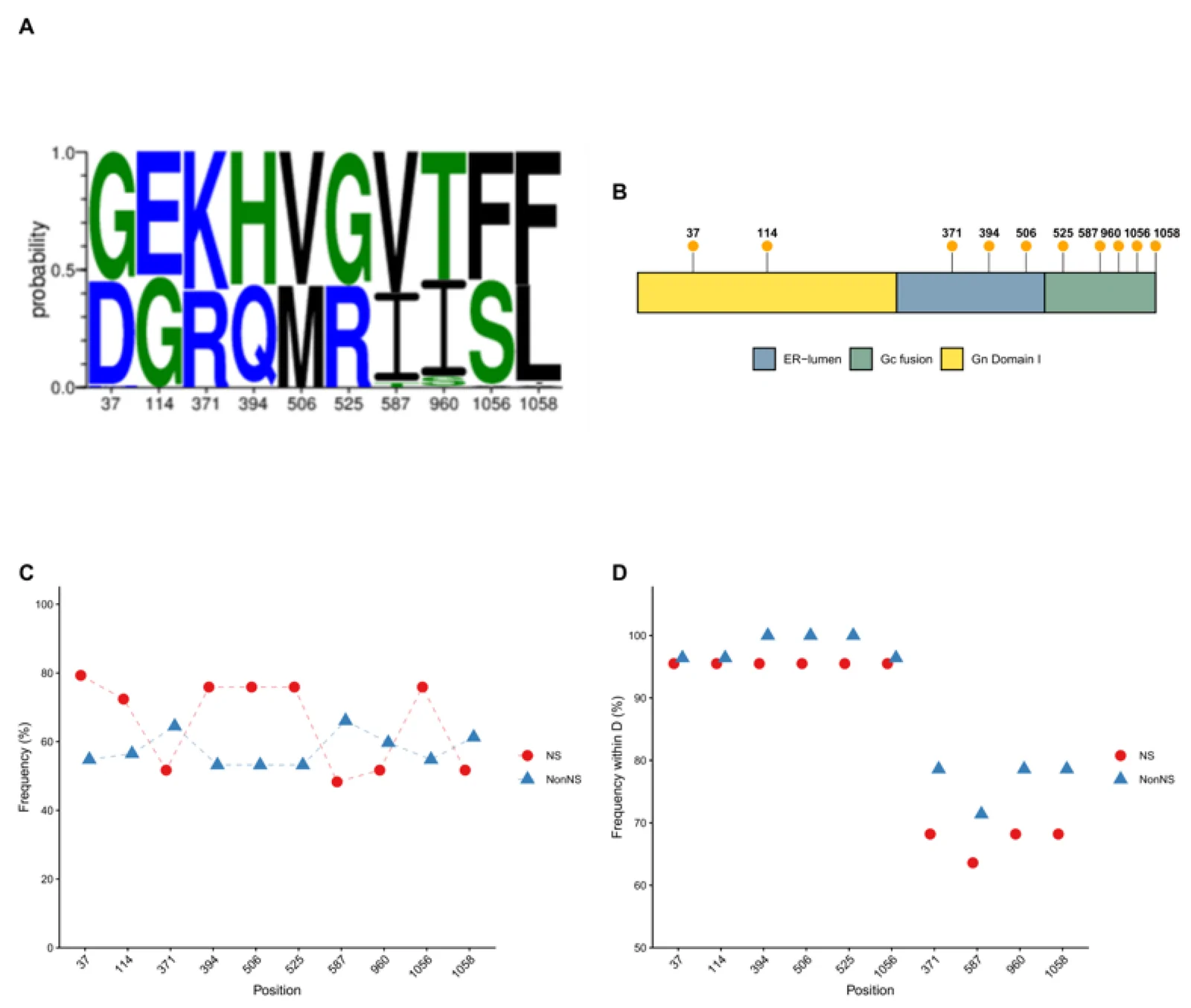

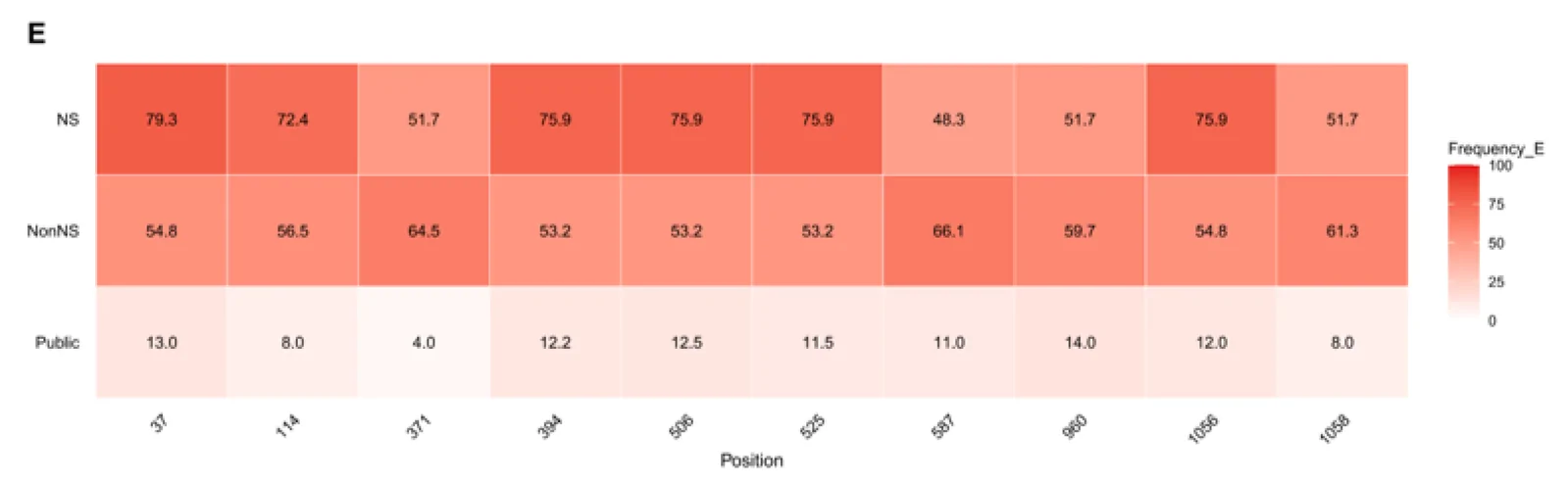
Signature-based identification and validation of candidate amino acid signatures. (**A**) WebLogo showing amino acid profile differences between NS and Non-NS groups at the 10 candidate sites. (**B**) Schematic diagram of Gn/Gc glycoprotein domains showing the locations of the 10 candidate sites. Positions 37 and 114 are located in Gn Domain I (receptor binding); positions 394, 506, and 525 in the ER-lumen domain (protein folding); and positions 960, 1056, and 1058 in the Gc fusion domain (membrane fusion). (**C**) Dot plot showing the frequency of NS-characteristic amino acids at each of the 10 candidate sites in the overall comparison (NS vs. Non-NS). (**D**) Dot plot showing the frequency of NS-characteristic amino acids restricted to genotype D only. No significant differences were observed between NS and Non-NS groups at any site (all *p* > 0.05). (**E**) Heatmap comparing the frequency of NS-associated amino acids across three datasets: public database (2219 sequences), Non-NS group, and NS group. In the public database, the NS-characteristic amino acids (red cells) are uniformly present at low frequencies (<15%), while Non-NS-characteristic amino acids dominate. The frequency gradient—public database (<15%) < Non-NS group (29–55%) < NS group (48–79%)—is evident across all 10 sites. Each cell represents the percentage of sequences carrying the indicated NS-characteristic amino acid at that position. The frequency gradient shown here should be interpreted with caution, as the public database lacks systematic genotype annotations. The gradient may partly reflect differential representation of genotype D sequences across datasets rather than providing independent evidence for NS-specific enrichment.

We performed systematic amino acid signature screening using an entropy-based algorithm to identify amino acid residues associated with neurological symptom status. The amino acid profile differences between the NS and Non-NS groups at the 10 candidate sites were visualized using WebLogo (Figure 3A). Mapping these sites to the Gn/Gc protein structure revealed distinct domain distribution patterns: positions 37 and 114 in Gn Domain I (receptor binding); positions 394, 506, and 525 in the ER-lumen domain (protein folding); and positions 960, 1056, and 1058 in the Gc fusion domain (membrane fusion) (Figure 3B). This domain distribution initially suggested potential functional relevance—these sites reside in regions critical for viral entry and membrane fusion. The frequency differences between the two groups at each of the 10 candidate sites are shown in Figure 3C. To address multiple comparisons, we performed Bonferroni correction across all 1073 positions (α’ = 4.66 × 10^−5^). No single site reached genome-wide significance, but the 10-site collective VESPA pattern remained highly significant, supporting these sites as genuine combinatorial signatures rather than false positives.

The complete list of candidate sites, including their positions, characteristic amino acids, and frequencies in each group, is summarized in Table 2. Of the ten sites, six (positions 37, 114, 394, 506, 525, and 1056) had NS characteristic frequencies ≥ 70%. The remaining four (positions 371, 587, 960, and 1058) showed frequencies between 48.3% and 51.7%, but were retained because their alternative residues dominated the Non-NS group (59.7–66.1%), maintaining a consistent directional difference between groups. All 10 sites were carried forward through the validation pipeline to determine whether they represented genuine phenotype-associated signals or genotype-specific background polymorphisms.

Given that all genotype F strains were from the Non-NS group, the differences observed in the overall comparison could be driven by genotype composition rather than by neurological symptom status per se. To test this, we performed a stratified analysis within genotype D. When analysis was restricted to genotype D (NS: 23, Non-NS: 28), no significant differences were observed at any of the 10 sites (all *p* > 0.05), with frequency differences all <11% and mostly <5% (Figure 3D). The detailed frequency comparison for each site within genotype D, including the exact *p*-values, is provided in Table 3. This result indicates that the initially observed differences were artifacts of genotype confounding rather than genuine phenotype associations.

We next performed public database validation using 2219 GP sequences from NCBI GenPept to assess whether these candidate sites represent common polymorphisms circulating in the general viral population. All NS-characteristic amino acids were uniformly low-frequency variants (<15%) in the natural SFTSV population (Figure 3E). This finding independently confirmed that these sites are not common polymorphisms but rather rare signatures specifically enriched in certain lineages, consistent with their role as genotype-specific markers rather than widespread functional determinants.

### 3.3. Genotype-Specific Signatures and Co-Occurrence Network

**Figure 4:**
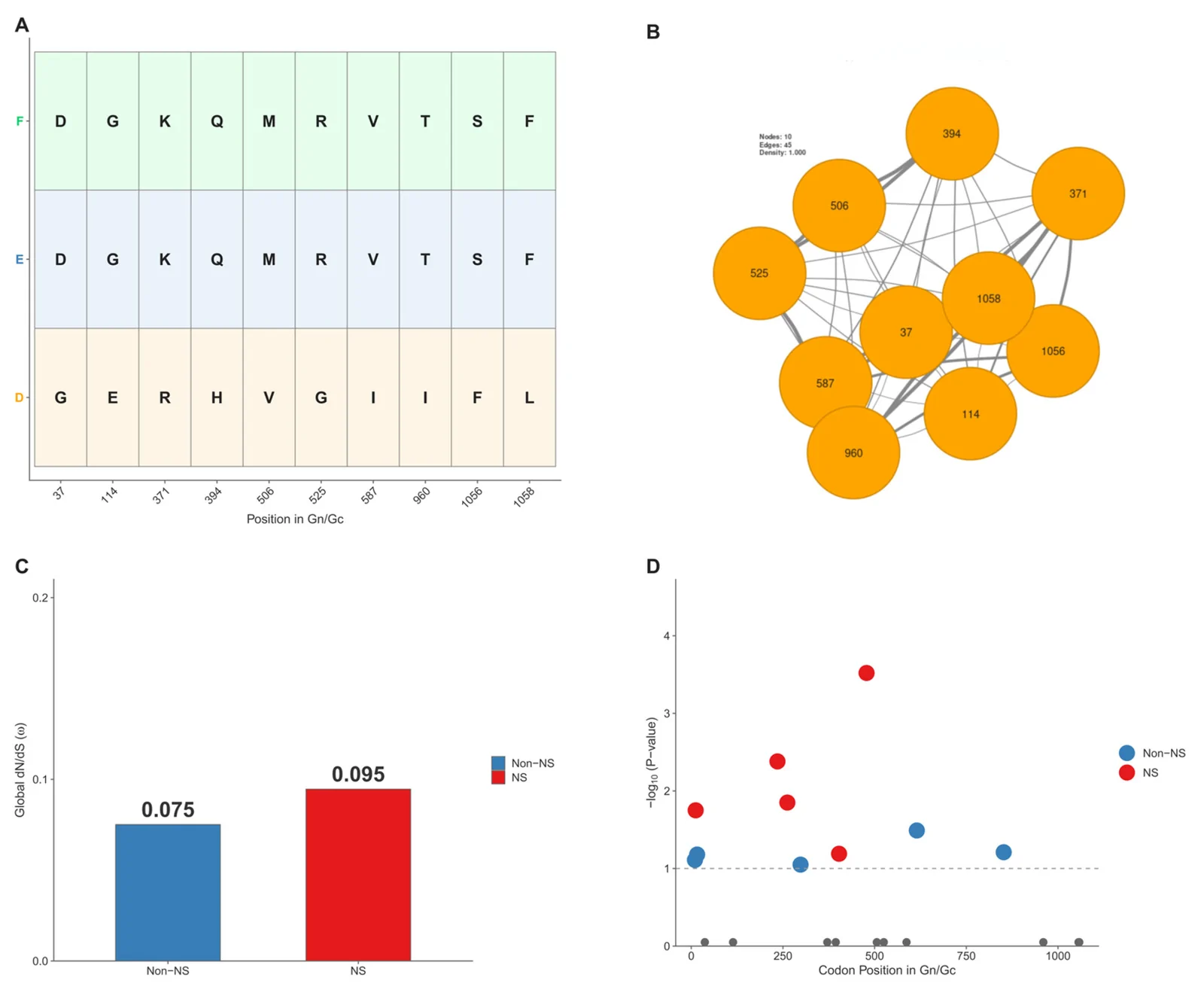
Genotype-specific molecular signatures of the 10 candidate sites. (**A**) Cross-genotype comparison of characteristic amino acid patterns at the 10 sites. Genotype D uniformly carries “GERHVGIIFL” (orange), while genotypes E and F carry “DGKQMRVTSF” (blue and green, respectively). (**B**) Co-occurrence network within genotype D (*n* = 51). Nodes represent the 10 amino acid positions; edges indicate significant co-occurrence pairs (Fisher’s exact test, *p* < 0.05), with edge thickness proportional to the strength of association. (**C**) Global dN/dS (ω) estimates for the NS (*n* = 29, ω = 0.0946) and Non-NS (*n* = 62, ω = 0.0751) groups. (**D**) MEME-detected positively selected sites; red and blue points represent NS and Non-NS groups, respectively. Gray triangles along the *x*-axis indicate the positions of the 10 candidate sites (37, 114, 371, 394, 506, 525, 587, 960, 1056, 1058), none of which were detected as positively selected by MEME.

Furthermore, cross-genotype comparison and co-occurrence network analysis were then performed to determine whether the 10 candidate sites were genotype-specific markers rather than phenotype-associated signals. Cross-genotype comparison revealed that genotype D strains uniformly carried the characteristic pattern “GERHVGIIFL” regardless of neurological symptom status (71–100% frequency), whereas genotypes E and F uniformly carried the distinct pattern “DGKQMRVTSF” (92–100%) (Figure 4A). Fisher’s exact test showed that the amino acid distribution at each of the 10 positions was significantly associated with genotype (all *p* < 0.0001). These findings indicate that the amino acid differences initially observed between NS and Non-NS groups were not driven by neurological symptom status itself, but rather reflected the underlying genetic background of the virus—specifically, the lineage-specific amino acid signatures that define genotype D versus genotypes E and F.

The 10-site combination showed 75.0% sensitivity and 100% specificity in distinguishing genotype D from E/F based on a reference set of 53 sequences spanning genotypes A–G, with geographic origins including China, Japan, and South Korea. These estimates are preliminary and derived from a limited reference set, and independent validation in geographically diverse datasets is required before any clinical application can be considered.

We further investigated the linkage among these 10 sites by performing co-occurrence network analysis on the 51 genotype D sequences. This analysis revealed that the 10 sites formed a fully connected network with 45 significant site pairs and a network density of 1.000, indicating that these sites are highly co-inherited within genotype D (Figure 4B). Two strong linkage modules were identified within this network: positions 394–506–525 and 371–960–1058, suggesting that these site clusters may be under shared evolutionary constraints. Within genotype D, positions 371, 587, 960, and 1058 formed a highly connected module; all pairwise co-occurrence frequencies reached 27.45% (*p* < 0.001). A weaker but still significant association was also observed between positions 37 and 114 (3.92%, *p* = 2.35 × 10^−3^). In contrast, no significant co-occurrence associations were detected in genotypes E or F, consistent with their highly conserved amino acid patterns. This stark contrast between genotypes further supports the interpretation that these sites are not flexible functional elements, but rather fixed genetic signatures that have been maintained during lineage diversification.

We performed genome-wide selection pressure analysis using four complementary methods (FEL, MEME, FUBAR, and SLAC) to assess whether the ten genotype-specific signatures might have functional relevance through adaptive evolution. Global purifying selection predominated across all viral proteins, with negative selection detected at 223 sites in Non-NS and 138 sites in NS (*p* ≤ 0.1). However, the NS group exhibited a higher global dN/dS ratio (ω = 0.0946) compared to the Non-NS group (ω = 0.0751) (Figure 4C), suggesting relatively relaxed purifying selection in NS-associated lineages—a condition that may facilitate the accumulation of lineage-specific substitutions over time. Cross-validated positively selected sites—defined as those detected by at least two methods—were identified at Codons 273, 12, and 13 (Table S3). Codon 273 was consistently detected by FEL, FUBAR, and SLAC in the Non-NS group, while Codons 12 and 13 showed positive selection signals in the NS group across multiple methods. MEME analysis additionally identified five positively selected sites in each group (NS: Codons 12, 235, 262, 403, and 478; Non-NS: Codons 10, 16, 298, 615, and 852), with Codon 478 showing the strongest signal exclusively in NS and Codon 615 detected in both groups (Figure 4D). Critically, none of the ten candidate sites identified through systematic signature screening overlapped with these cross-validated positively selected sites. Codon 37, one of the candidate sites, was detected by FUBAR alone (posterior probability > 0.9) but did not meet the cross-validation criterion, indicating that the candidate signatures are not direct targets of positive selection but rather lineage-specific background polymorphisms.

## 4. Discussion

In this study, we set out to investigate whether specific amino acid variations in the SFTSV Gn/Gc glycoprotein, initially identified as statistically associated with neurological symptoms, represent genuine functional determinants of neuropathogenesis or merely reflect underlying viral genotype background. Through sequential analyses encompassing M segment-based phylogenetic reconstruction, public database validation, genotype-stratified analysis, cross-genotype comparison, co-occurrence network analysis, and selection pressure inference, we systematically deconstructed this association. Our findings demonstrate that the ten Gn/Gc sites initially linked to neurological symptoms are, in fact, genotype D-specific molecular signatures that have been maintained through lineage diversification, rather than direct viral determinants of neurological disease. This study serves as a cautionary example for virus phenotype association studies and highlights the importance of distinguishing genotype-specific markers from functional determinants.

The initial systematic amino acid signature screening identified ten Gn/Gc sites as candidate markers distinguishing neurological symptom from non-neurological symptom groups, with several sites mapping to functionally critical domains. Positions 37 and 114 are located within Gn Domain I, which is responsible for receptor binding. Positions 394, 506, and 525 reside in the ER lumen domain, which is involved in protein folding. Positions 960, 1056, and 1058 are situated in the Gc fusion domain, which mediates membrane fusion. This domain distribution initially suggested plausible biological relevance, as the Gn/Gc glycoproteins are the primary determinants of viral entry and tissue tropism [20]. Recent studies have demonstrated that specific mutations in the SFTSV glycoprotein—identified through targeted mutagenesis at sites distinct from our candidate positions—can modulate viral entry and immune escape, with distinct amino acid substitutions affecting receptor binding affinity, membrane fusion activity, and susceptibility to neutralizing antibodies [21]. However, these functionally validated mutations were identified through targeted mutagenesis rather than natural sequence association, and the reported sites are different from the 10 positions identified in our VESPA screening. This highlights a fundamental challenge in viral pathogenesis research: statistical correlation in clinical isolates does not necessarily imply functional causation, particularly when the viral population is structured by genotype [22]. Critically, our selection pressure analysis showed that none of the 10 candidate sites are under positive selection, further distinguishing them from the functionally validated mutations reported in these studies.

The decisive evidence that refuted the functional interpretation came from our genotype-stratified analysis. When the association analysis was restricted to M segment-defined genotype D, which is the predominant genotype in our dataset and the one driving the initial signal, all ten candidate sites showed no significant frequency differences between neurological symptom and non-neurological symptom groups (all *p* > 0.05). Frequency differences were uniformly below 11% and mostly below 5%. This complete attenuation of signal upon stratification represents a classic hallmark of confounding. It demonstrates that the observed “neuro signature” was an epiphenomenon of the uneven distribution of genotype D between the two clinical groups rather than a genuine phenotype-associated signal. This scenario mirrors well-documented examples in other viral systems where phylogenetic structure, if not properly accounted for, can create spurious genotype–phenotype associations [23].

Public database validation further substantiated this conclusion. Analysis of 2219 glycoprotein precursor sequences from the NCBI GenPept database revealed that all NS characteristic amino acids were uniformly low-frequency variants (<15%) in the global SFTSV population, ruling out their role as common functional polymorphisms. A clear frequency gradient was observed: the frequency increased from <15% in the public database to 29–55% in the Non-NS group and 48–79% in the NS group. This pattern is precisely what would be expected if the signal were driven by genotype D enrichment rather than neurological symptom status. This observation is consistent with the understanding that many lineage-defining polymorphisms in RNA viruses represent neutral or nearly neutral hitchhikers that have been carried along during lineage diversification rather than direct targets of adaptive evolution [24]. Nevertheless, a limitation of this public database validation should be acknowledged: the retrieved GP sequences lacked detailed background information, including geographical origin, sampling time, host type, and genotype composition. Thus, the observed low frequency (<15%) of NS-characteristic amino acids may primarily reflect the limited representation of genotype D sequences in the database rather than genuine rarity of these residues in nature. This interpretation is consistent with the view that these sites are lineage-specific markers rather than functional determinants, a conclusion independently supported by the genotype-stratified and cross-genotype analyses performed on our dataset. Nonetheless, the public database results remain consistent with this conclusion.

Cross-genotype comparison further confirmed that these ten sites are genotype-specific markers rather than phenotype-associated signals. Genotype D strains uniformly carried the characteristic pattern “GERHVGIIFL” regardless of neurological symptom status (71–100% frequency), whereas genotypes E and F uniformly carried the distinct pattern “DGKQMRVTSF” (92–100%). The near-perfect segregation of this motif highlights a deep evolutionary divergence in the M segment and provides a clear diagnostic signature that distinguishes these lineages. Notably, the 10-site combination demonstrated 75.0% sensitivity and 100% specificity for genotype D identification. This offers a practical molecular tool for rapid genotyping without the need for full genome sequencing. This approach is particularly valuable in resource-limited settings where whole-genome sequencing may not be feasible, yet genotype D surveillance is clinically relevant given its association with higher case fatality rates in previous studies [25].

We acknowledge limitations of the 10-site marker panel. First, the performance estimates were derived from a limited reference set (*n* = 53) and have not been externally validated; the 100% specificity may decrease with broader sampling. Second, this marker has only been evaluated in our Shandong dataset and requires validation in independent datasets from other endemic regions. Third, its clinical applicability remains preliminary and requires further assessment in prospective studies before practical use. We emphasize that this finding should be viewed as a proof-of-concept observation rather than a validated diagnostic tool.

The co-occurrence network analysis provided further insight into the linkage structure among these sites. The ten sites formed a fully connected network with 45 significant site pairs and a network density of 1.000 within genotype D, indicating that these sites are highly co-inherited within genotype D. This pattern is consistent with linkage networks described in other viral systems, where physically or functionally interacting residues evolve in a coordinated manner to maintain protein structural integrity and have been shown to predict disease outcomes [26,27]. The tight co-occurrence network we observed within genotype D, combined with the absence of such networks in genotypes E and F, suggests that these sites are not flexible functional elements but rather fixed genetic signatures maintained during lineage diversification.

From an evolutionary perspective, the absence of positive selection at the ten candidate sites is a critical observation. Our selection pressure analysis employed four complementary methods (FEL, MEME, FUBAR, and SLAC). It identified cross-validated positively selected sites at Codons 273, 12, and 13. However, none of the ten signature-identified sites overlapped with these robust signals. Codon 37, one of the candidate sites, was detected by FUBAR alone but did not meet the cross-validation criterion of detection by at least two methods. This illustrates the importance of stringent validation in selection pressure inference. The global dN/dS ratio was slightly higher in the NS group (ω = 0.0946) than in the Non-NS group (ω = 0.0751). However, given that NS cases are predominantly genotype D (79.3%), this difference likely reflects the evolutionary background of genotype D rather than phenotype-associated relaxed selection. Bootstrap resampling analysis (10,000 replicates) based on the FUBAR output yielded overlapping 95% confidence intervals between the two groups (NS: 0.071–0.118; Non-NS: 0.056–0.094), indicating that the observed difference is not statistically significant. Critically, none of the 10 VESPA-identified sites overlapped with MEME-detected positively selected sites, and no positive selection signals were detected at these sites across any of the four independent methods. The consistent absence of positive selection signals across multiple methods confirms that these sites are passive markers rather than active drivers of neuropathogenesis. We acknowledge that codon-based selection detection methods were originally developed for populations with simpler demographic histories, and their assumptions may not fully capture the highly structured nature of SFTSV populations. Nevertheless, the consistent results across four independent methods support the robustness of this conclusion.

The geographic distribution of SFTSV genotypes in East Asia is markedly uneven: genotype D predominates in Shandong, whereas genotypes E and F are more common in Japan and South Korea. Under an updated genome-wide genotyping framework, genotype IV (corresponding to conventional genotype D) is the predominant lineage in China, while genotype X dominates in Japan (88.8% prevalence). These distinct genotype distributions may partly contribute to the observed international differences in case-fatality rates (CFR).

From a clinical epidemiological perspective, the exclusive presence of genotype F in the Non-NS group warrants careful interpretation. While this pattern suggests a potential association between genotype F and reduced neurological involvement, the statistical phenomenon of complete separation renders the calculated odds ratio (OR = 0.063) unstable and likely inflated. In our dataset, genotype F perfectly predicts Non-NS status. This is a well-recognized issue in logistic regression when an exposure variable perfectly predicts the outcome [28]. We therefore interpret this as an associative trend rather than a robust protective effect. This finding should be considered a hypothesis-generating observation that warrants validation in independent datasets with more balanced genotype distributions. The association remained significant in Firth’s penalized logistic regression sensitivity analysis (Firth OR = 0.086, 95% CI: 0.009–0.383, *p* = 0.0005), confirming its robustness despite the complete separation issue. Nevertheless, the OR should still be interpreted with caution given the small sample size. This observation is consistent with previous reports showing differential virulence among SFTSV genotypes, where genotype D has been linked to elevated case fatality rates and stronger inflammatory responses, while genotype F has been linked to relatively lower mortality in both human studies and animal models. Given the limited sample size of genotype F in our dataset (*n* = 18), this finding remains preliminary. The biological basis of this potential trend requires further investigation through functional studies and independent epidemiological validation [29].

However, genotype distribution alone cannot fully explain these CFR differences. Host demographic factors, particularly population aging, are well-established risk factors for SFTS mortality, yet Japan and South Korea have older populations than China, while their reported CFRs are generally not higher—suggesting that surveillance practices may offset demographic effects. Surveillance bias, including differences in case definitions, diagnostic capacity, and reporting thresholds for mild versus severe cases, may also substantially influence CFR estimates across countries [30,31].

Several limitations should also be acknowledged. Additionally, due to the retrospective nature of this study, systematic information on antiviral treatments (e.g., ribavirin, favipiravir) and detailed operational procedures for neurological assessment were not available. This limitation should be considered when interpreting the associations. Although the NS and Non-NS groups were comparable in baseline characteristics, we cannot rule out the possibility that differences in antiviral treatment may have influenced the occurrence or severity of neurological symptoms. Furthermore, the limited number of recombinant sequences (*n* = 8) precludes a formal assessment of whether their exclusion may have influenced the genotype–phenotype associations. However, based on the available data, their clinical and genotypic profiles did not differ significantly from the non-recombinant group, suggesting that any potential bias, if present, is unlikely to be substantial. The retrospective nature of this study also resulted in incomplete documentation of underlying diseases (missing in 56/91 cases), which precluded their inclusion as covariates in the multivariable model. This limitation should be considered when interpreting the adjusted associations between genotype and neurological symptoms. Additionally, post hoc power analysis based on the maximum observed frequency difference within genotype D (10.4%) indicated that the current sample size (NS = 23, Non-NS = 28) provided only 13.5% power to detect this difference (α = 0.05). To achieve 80% power, approximately 281 cases per group would be required. Therefore, the lack of significant differences within genotype D should be attributed to insufficient statistical power rather than interpreted as evidence of no biological association. Larger studies are needed to further validate these findings. Despite these limitations, the stepwise validation framework established here provides a practical approach for distinguishing genotype-specific background polymorphisms from functional determinants in future virus–phenotype association studies.

In conclusion, this study resolves an apparent virus phenotype association as a classic example of genotype confounding, identifying ten Gn/Gc amino acid sites as reliable lineage-specific markers for M segment-defined genotype D rather than functional determinants of neurological symptoms. This work reinforces the principle that phenotypic associations in viral genomic epidemiology must be stringently tested for stratification before being interpreted as functional. The distinct genetic and clinical features of SFTSV genotypes, particularly D and F, highlight the need for continued molecular surveillance and further investigation of the interplay between viral genetics, host immunity, and clinical outcome. As SFTSV continues to spread across East Asia and the WHO has listed it as a priority pathogen, understanding the true nature of genotype–phenotype associations will be essential for accurate risk assessment and the development of targeted surveillance and intervention strategies. Distinguishing genuine functional determinants from genetic background noise is a critical step toward this goal.

## Figures and Tables

**Table 1:** Baseline characteristics of SFTS patients stratified by neurological symptoms and M segment genotypes.

Characteristic	Non-NS (*n* = 68)	NS (*n* = 31)	*p*-Value
Age (years), median (IQR)	67 (61, 72)	70 (67, 75)	0.042
Time from onset to admission (days), median (IQR)	5.0 (5.0, 7.0)	7.0 (5.0, 8.0)	0.217
Sex, *n* (%)			0.781
Male	35 (51.5%)	18 (58.1%)	
Female	33 (48.5%)	13 (41.9%)	
Underlying diseases, *n* (%)			1
Yes	8 (11.8%)	5 (16.1%)	
No	17 (25.0%)	12 (38.7%)	
Unknown	43 (63.2%)	14 (45.2%)	
WBC (×10^9^/L), median (IQR)	2.56 (1.50, 4.24)	2.91 (2.05, 6.89)	0.411
AST (U/L), median (IQR)	455 (279, 782)	458 (200, 835)	0.936
ALT (U/L), median (IQR)	190 (120, 252)	199 (117, 245)	0.946
PLT (×10^9^/L), median (IQR)	47 (33, 62)	52 (38, 70)	0.501

Data are presented as median (IQR) for continuous variables and *n* (%) for categorical variables. *p*-values were calculated using Wilcoxon rank-sum test for continuous variables and Fisher’s exact test for categorical variables as appropriate. Abbreviations: NS, neurological symptoms; Non-NS, non-neurological symptoms; WBC, white blood cell count; AST, aspartate aminotransferase; ALT, alanine aminotransferase; PLT, platelet count. Data on underlying diseases were incomplete (43 missing in Non-NS, 14 missing in NS).

**Table 2:** Signature amino acid sites in the Gn/Gc glycoprotein distinguishing NS from Non-NS groups.

Position	NS Characteristic AA	NS Frequency (%)	Non-NS Characteristic AA	Non-NS Frequency (%)	Fisher *p*-Value
37	G	79.3	D	54.8	0.0016
114	E	72.4	G	56.5	0.0133
371	R	51.7	K	64.5	0.5427
394	H	75.9	Q	53.2	0.0058
506	V	75.9	M	53.2	0.0058
525	G	75.9	R	53.2	0.0058
587	I	48.3	V	66.1	0.5427
960	I	51.7	T	59.7	0.5427
1056	F	75.9	S	54.8	0.0027
1058	L	51.7	F	61.3	0.5427

AA, amino acid. All NS-characteristic amino acids were present at <15% frequency in the public database (*n* = 2219). Positions 371, 587, 960, and 1058 (*p* > 0.05) were retained based on high discriminatory weight in the initial screening.

**Table 3:** Frequency comparison of NS-associated amino acids within genotype D.

Position	NS Characteristic AA	NS Frequency (*n* = 23)	Non-NS Frequency (*n* = 28)	Frequency Difference (%)	*p*-Value
37	G	95.50%	96.40%	0.9	1
114	E	95.50%	96.40%	0.9	1
394	H	95.50%	100%	4.5	0.458
506	V	95.50%	100%	4.5	0.458
525	G	95.50%	100%	4.5	0.458
1056	F	95.50%	96.40%	0.9	1
371	R	68.20%	78.60%	10.4	0.521
587	I	63.60%	71.40%	7.8	0.761
960	I	68.20%	78.60%	10.4	0.521
1058	L	68.20%	78.60%	10.4	0.521

Data are presented as percentages. *p*-values were calculated using Fisher’s exact test.

## Data Availability

The viral genome sequences generated in this study have been deposited in GenBank under accession numbers PZ831492–PZ831590. These sequences are currently under embargo until the manuscript is accepted for publication. Upon acceptance, the sequences will be made publicly accessible. The clinical data supporting the findings of this study are not publicly available due to patient privacy restrictions, but are available from the corresponding authors upon reasonable request.

## Supplementary Materials

The following supporting information can be downloaded at: https://www.mdpi.com/article/10.3390/pathogens15090946/s1, Table S1: primers used for nested RT-PCR amplification of the SFTSV M segment; Table S2: clinical characteristics of recombinant vs. non-recombinant sequences; Table S3: positively selected sites identified by four selection pressure methods. Figure S1. Schematic representation of recombination events identified in the SFTSV M segment. Eight recombinant sequences were identified among the 99 M-segment sequences. Each recombinant is shown as a horizontal bar, with regions derived from different parental genotypes (A–F) distinguished by colors according to the legend. Vertical dashed lines indicate recombination breakpoints, with numbers above or below denoting the nucleotide positions in the M segment. Asterisks denote breakpoints that were refined by SimPlot analysis. The parental genotypes contributing to each recombinant sequence are indicated on the left.

## Abbreviations

SFTS	Severe fever with thrombocytopenia syndrome
SFTSV	Severe fever with thrombocytopenia syndrome virus
NS	Neurological symptoms
Non-NS	Non-neurological symptoms
GP	Glycoprotein precursor
Gn	Glycoprotein N
Gc	Glycoprotein C
VESPA	Viral Epidemiologic Signature Pattern Analysis
RDP	Recombination Detection Program
FUBAR	Fast Unconstrained Bayesian AppRoximation
MEME	Mixed Effects Model of Evolution
FEL	Fixed Effects Likelihood
SLAC	Single-Likelihood Ancestor Counting
OR	Odds ratio
CI	Confidence interval
