# Supplementary material for: Genotype-Specific Amino Acid Signatures in the Gn/Gc Glycoprotein of Severe Fever with Thrombocytopenia Syndrome Virus Associated with Neurological Symptoms
Source: Pathogens. 2026 Sep 6;15(9):946. doi: 10.3390/pathogens15090946 (PMC13610127; doi:10.3390/pathogens15090946)
Supplement: Supplementary file 1 [file pathogens-15-00946-s001.zip › FigureS1.pdf]

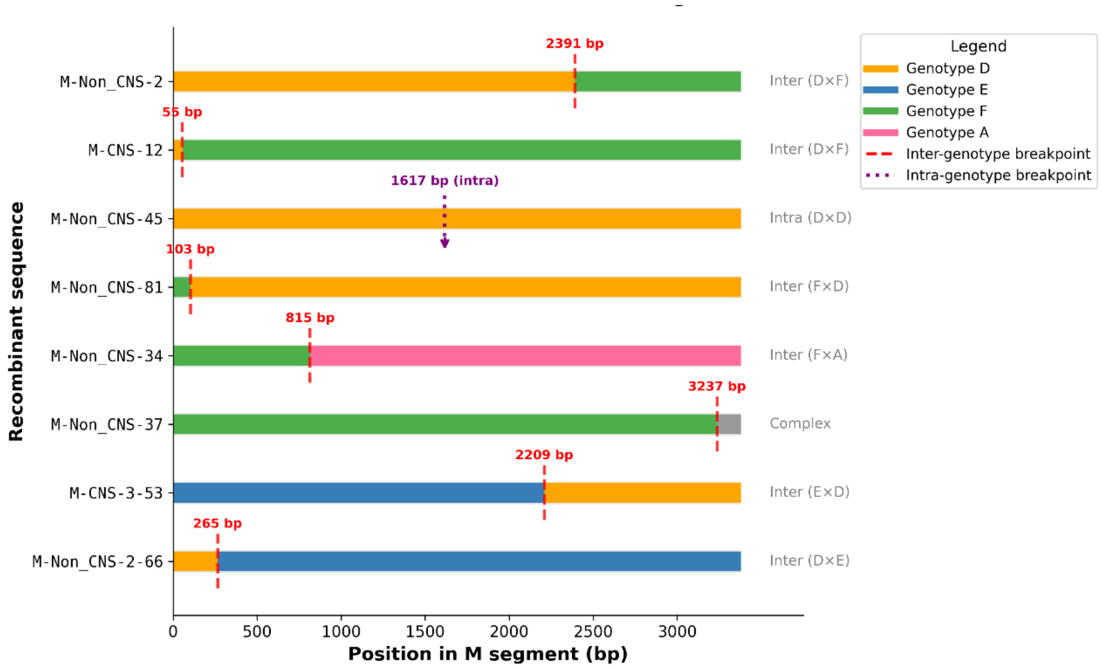

Figure S1. Schematic representation of recombination events identified in the SFTSV M segment. Eight recombinant sequences were identified among the 99 M-segment sequences. Each recombinant is shown as a horizontal bar, with regions derived from different parental genotypes (A–F) distinguished by colors according to the legend. Vertical dashed lines indicate recombination breakpoints, with numbers above or below denoting the nucleotide positions in the M segment. Asterisks denote breakpoints that were refined by SimPlot analysis. The parental genotypes contributing to each recombinant sequence are indicated on the left.
