# Supplementary material for: Genotype-Specific Amino Acid Signatures in the Gn/Gc Glycoprotein of Severe Fever with Thrombocytopenia Syndrome Virus Associated with Neurological Symptoms
Source: Pathogens. 2026 Sep 6;15(9):946. doi: 10.3390/pathogens15090946 (PMC13610127; doi:10.3390/pathogens15090946)
Supplement: Supplementary file 1 [file pathogens-15-00946-s001.zip › supplementary tables.pdf]

Table S1. primers used for nested RT-PCR amplification of the SFTSV M segment

Round	Fragment	Primer name	Sequence (5′→3′)	Amplicon (bp)
1st	Gn-1	FGn-1	ATGATGAAAGTCATCTGGTTCTCCT	~832
		RGn-1	CACTGGTCTTGCAAGTCTTTTCTT	
	Gn-2	FGn-2	AGGGACTGGGCCTTGTTCTG	~851
		RGn-2	ATGCCTTACCCTTCTAGGTCTTGC	
	Gc-1	FGc-1	ACCAGGATGATGTTAGGATTGAGAT	~951
		RGc-1	CTTAGAGTAGCACACAGCGTCATCC	
	Gc-2	FGc-2	ACCTCGTGGGTATAGAGCTACGA	~738
		RGc-2	AGCCAGCTTCGTCCTCGATC	
2nd (nested)	Gn-1	FSGn-1	TCATCTGGTTCTCCTCTCTGATCTG	~812
		RSGn-1	ACAAGCCTTTTCTTCTGATTCAGAA	
	Gn-2	FSGn-2	AGGGACTGGACCTTGTTCTGAA	~831
		RSGn-2	CTTCTGGGTCTTGCCATCTCA	
	Gc-1	FSGc-1	TGTTAGGATTGAGATGGCAAGACC	~931
		RSGc-1	CACACAGCGTCATCCACTCG	
	Gc-2	FSGc-2	TATAGAGCTACGAGTGGATGACGC	~718
		RSGc-2	GTCCTCGATCGAAATACCTGTTTT	

All primers were used at an annealing temperature of 58°C. First-round amplicons ranged from approximately 738 to 951 bp, and second-round (nested) amplicons ranged from approximately 718 to 931 bp.

Table S2. clinical characteristics of recombinant vs. non-recombinant sequences

Characteristic	Recombinant (n=8)	Non-recombinant (n=91)	P-value
Neurological symptoms	2 (25.0%)	29 (31.9%)	1
Age (years)	66 (58, 71)	68 (62, 73)	0.542
Male sex	5 (62.5%)	53 (58.2%)	1
Genotype D	5 (62.5%)	51 (56.0%)	0.735
Genotype F	2 (25.0%)	18 (19.8%)	0.662

P-values calculated by Fisher's exact test (categorical) or Wilcoxon rank-sum test (continuous). No significant differences were observed in any clinical or genotypic parameter between recombinant and non-recombinant sequences, suggesting that

exclusion of recombinant sequences did not introduce systematic bias in the genotype-phenotype association analysis.

Table S3. positively selected sites identified by four selection pressure methods

Group	Method	Positively selected sites
Non-NS	FEL	273
	MEME	10, 16, 298, 615, 852
	FUBAR	37, 273
	SLAC	273
NS	FEL	13
	MEME	12, 235, 262, 403, 478
	FUBAR	12, 13, 37
	SLAC	None

SLAC, FEL, MEME significance threshold: $P < 0.1$; FUBAR significance threshold: posterior probability >0.9. Sites detected by at least two methods (Codons 273, 12, and 13) were considered cross-validated positively selected sites.
